# Implementing personalized medicine with asymmetric information on prevalence rates

**DOI:** 10.1186/s13561-016-0113-7

**Published:** 2016-08-18

**Authors:** Fernando Antoñanzas, Carmelo A. Juárez-Castelló, Roberto Rodríguez-Ibeas

**Affiliations:** Department of Economics, University of La Rioja, Cigüeña 60, 26006 Logrono, Spain

**Keywords:** Endogenous reference price, Exogenous reference price, Off-patent drug, Generic drug, Pharmaceutical expenditures, I11, I18, L13, L51

## Abstract

Although personalized medicine is becoming the new paradigm to manage some diseases, the economics of personalized medicine have only focused on assessing the efficiency of specific treatments, lacking a theoretical framework analyzing the interactions between pharmaceutical firms and healthcare systems leading to the implementation of personalized treatments. We model the interaction between the hospitals and the manufacturer of a new treatment as an adverse selection problem where the firm does not have perfect information on the prevalence across hospitals of the genetic characteristics of the patients making them eligible to receive a new treatment. As a result of the model, hospitals with high prevalence rates benefit from the information asymmetry only when the standard treatment is inefficient when applied to the patients eligible to receive the new treatment. Otherwise, information asymmetry has no value. Personalized medicine may be fully or partially implemented depending on the proportion of high prevalence hospitals.

## Background

Since human genome decoding in 2000, the fast development of genomics and other biosciences has allowed new approaches to diagnose and manage diseases, tailoring protocols and treatments to personal and medical characteristics of patients, and thereby eliminating ineffective therapies based in the traditional trial and error clinical paradigm [1]. The term personalized medicine, first used by Gibson [2] in 1971, designs the new healthcare paradigm based on genomics to match new treatments and patients. This term has evolved in time, and nowadays there are several definitions and interpretations [3]. Throughout this paper, personalized medicine or “stratified medicine” refers to the practice “where therapies are matched with specific patient population characteristics using clinical biomarkers” [4]. The general term “personalized medicine” has also been used as a synonymous of “pharmacogenomics” to highlight the evaluation of genetic variations to identify patients likely to respond to specific therapies [5].

The application of personalized medicine requires to have instruments (tests) to stratify patients as well as personalized treatments [6]. The selection of the optimal therapy depends on the patient’s genetic characteristics, as the presence or absence of a biomarker usually predicts the response to a particular treatment. Until recently, there were no mechanisms to stratify the population of patients on the basis of a genetic biomarker, thus precluding the use of specific treatments, if available. The new possibilities offered by genomics (there were over 1,800 genetic tests targeting different genome sections related to known conditions in 2012 as described in Antoñanzas et al. [7]; today, a casual observation of different genetic databases shows that the number of tests has skyrocketed up to more than 15,000 tests for more than 2,800 genes) provide healthcare systems with a highly reliable tool to stratify patients, and prescribe the best treatment. For example, there are diagnostic tests based on genetics (Oncotype DX® and MammaPrint®) that place breast cancer women into different risks categories for which there are specific treatments. Breast cancer women with an overexpression of HER2 may receive chemotherapy plus Herceptin® (trastuzumab) instead of only chemotherapy, thus reducing the recurrence of the tumor (as described by Hornberger et al. [8]). Likewise, there are several drugs available to treat colorectal cancer, although their efficacy differ across the genetic mutation involved in the development of the disease. For example, Erbitux® (cetuximab) and Vectibix® (panitumumab) are only recommended for patients with the normal expression of the KRAS gene (see for instance Paik et al. [9]; Lièvre et al. [10]; Behl et al. [11]; Thierry et al. [12]).

For health authorities, personalized medicine may help improve health outcomes and save costs, mostly derived from no administering some drugs to patients anticipated as being unresponsive, although certain personalized treatments may also increase costs. Thus, the implementation of personalized medicine requires that health decision makers assess the value of this type of medicine compared to the standard treatments for every specific indication and medical context, as the incremental health benefits of personalized medicine usually demand higher health budgets too. At the same time, pharmaceutical firms need economic incentives (high enough investment returns) to develop personalized drugs. Pricing and reimbursement policies become relevant, and play a fundamental role in providing such returns to investments [13]).

Regardless of the health insurance system, once a new treatment is authorized by a central health authority, the decision to apply it corresponds to each individual health provider (e.g. a hospital). Such decision will depend on several variables such as the price for the new treatment set by the pharmaceutical firm, the quality of the treatments, the reimbursement mechanism to health providers, as well as on the particular characteristics of the patients. As this set of information may not be necessarily known by all parties involved (the health providers and the firm), the price of the new treatment may differ across health providers, and personalized medicine may be fully or partially implemented in the health system. As it will be described later, this paper analyzes these issues with a simple adverse selection model.

Until now, research on the economics of personalized and predictive medicine has mainly focused on economic evaluations assessing the efficiency of specific tests to make decisions on price, reimbursement and disease management (see Payne and Annemans [14]; Payne et al. [15]; Meckley and Neumann [16]) as well as on the efficiency of individualized medicine [17, 18], although there are also some papers dealing with methodological issues in this area as Postma et al. [19] and Annemans et al. [20].

However, there are few theoretical publications modelling the decision making processes related to personalized medicine. Sahlin [21] discussed the ethical, economic and regulatory problems raised by the application of personalized medicine without modelling the decision-making. Chiappori [22] analyzed the welfare effects of predictive medicine from an insurance perspective, and focused on estimating the “Hirschleifer effect”, whereby more information available might reduce welfare. Bardey and De Donder [23] framed the individual decision to take a genetic test whose results may influence the conditions of health insurance in a context of moral hazard. Garrison and Finley [13] analyzed the role of some economic elements (among them, the reimbursement system and the diagnostic intellectual property) in the development of personalized medicine. Antonanzas et al. [24] studied the conditions under which genetic tests would be used by a health authority as a previous step to implement personalized medicine. However, these papers lack an integrated framework to analyze the decision to implement personalized medicine.

This paper analyzes from a theoretical perspective the decision making processes dealing with personalized health care by providing an integrated framework that combines some epidemiological, clinical and economic variables. In particular, we focus on the decision to adopt personalized medicine hospitals face when they do not have perfect information about the best treatment for a population of patients with a given disease. They may use, at a cost, a test to match patients with treatments, and therefore, to make a better-informed decision. As the tests do not perfectly classify the screened population, there is a trade-off between the efficacy of the medical procedures and their costs (test included) that may advice on efficiency grounds in favor or against the utilization of personalized medicine. A manufacturer sells a new treatment suitable for some patients, and hospitals must decide whether to adopt personalized medicine. We model the interaction between the hospitals and the manufacturer as an adverse selection problem where the manufacturer does not have perfect information on the prevalence across hospitals of the condition suitable to receive the new treatment, and state the conditions under which personalized medicine is implemented.

As it is well known from registries, the prevalence of any disease is usually different across regions and hospitals. When referred to the prevalence of some genetic patterns of specific conditions (e.g. tumors), such prevalence rates, as shown by the biomarkers used to identify them, may also be different across hospitals. One of the reasons for these differences is that patients can be exposed to different environments (pollution, poverty, working conditions, etc.). Dissimilar prevalence rates may also be caused by unknown factors (not yet discovered by science, and correlated to those rates) affecting patients clustered across hospitals differently. Information on the prevalence rates of the genetic characteristics of the patients in the whole population is generally of public domain; however, at micro level, hospitals have private information about their own prevalence rate while the manufacturer of the new treatment has usually no access to such prevalence and only knows the average prevalence rate.

The paper is organized as follows. In section Methods we describe the model and characterize the optimal prices for the personalized treatment under perfect and asymmetric information. The conditions under which personalized medicine is implemented are analyzed. We discuss the results in section Discussion and present the conclusions in section Conclusions.

## Methods

### The model

We consider a pool of hospitals dealing with patients diagnosed with a given disease. Without loss of generality, we assume that each hospital treats a population of patients whose size is normalized to one; there are two types of patients (type 1 and 2) who differ in their response to the available treatments due to their genetic characteristics, with *π* > 0 being the proportion of type 1 patients. Hospitals differ in the prevalence rate (proportion) of type 1 patients. We assume that the prevalence *π* may be low (*π*_*l*_) or high (*π*_*h*_), with 1 > *π*_*h*_ > *π*_*l*_ > 0, being *q* ∈ (0, 1) the proportion of hospitals with high prevalence and $\overline{\pi}=q{\pi}_h+\left(1-q\right){\pi}_l$ the population average prevalence.

Each patient does not know her type, and neither does the hospital that treats her, although the distribution of types (e.g. the prevalence of each type) is known by each hospital. There are available a new treatment (treatment 1) and a standard treatment (treatment 2). Let *p*_*ij*_ be the probability that the treatment *j* is successful when applied to a patient of type *i*, *i* = 1, 2; *j* = 1, 2:

$$ {p}_{ij}=\left\{\begin{array}{c}\hfill 1\  if\ i=j\hfill \\ {}\hfill \alpha\ if\ i=1,j=2\hfill \\ {}\hfill \beta\ if\ i=2,j=1\hfill \end{array}\right. $$

The standard treatment is successful for type 2 patients and the new treatment for type 1 patients. However, type 1 patients get cured with a probability *α* ∈ [0, 1) when they receive the standard treatment, and type 2 patients get cured with a probability *β* ∈ [0, 1) with the new treatment. When a treatment is successful, the patient gets a benefit *b* > *d*, where *d* ≥ 0 is the disutility if either the patient is left untreated or the treatment is not successful. The expected benefit type *i* patient gets when she receives treatment *j* is *p*_*ij*_*b* − (1 − *p*_*ij*_)*d*, *i* = 1, 2, *j* = 1, 2. The standard treatment costs *C* per patient. We assume that *b* > *C*. Benefit *b* can be interpreted either as the economic value of the QALYs or as the number of QALYs (in this case, the economic value of each QALY is assumed to be equal to 1) derived from successful treatments.

For instance, we may think of the population of women with breast cancer, some of them having tumors with an overexpression of the HER2/neu-receptor (type 1 patients). The new treatment may consist of the application of a new treatment combining chemotherapy and monoclonal antibodies (trastuzumab) while the standard treatment applies only chemotherapy. Women with an overexpression of the HER2/neu-receptor need to be given monoclonal antibodies besides chemotherapy as chemotherapy alone does not have any effect on their health (in this case, *α* would be equal to zero). Chemotherapy works for women with no overexpression of the HER2/neu-receptor. If monoclonal antibodies are also prescribed to these women, they are cured also with probability 1 (in this case, *β* would be equal to 1). The values of *α* and *β* may differ for other conditions.

The idea behind personalized medicine is to give each patient the best treatment according to her genetics and medical characteristics. In the process, resources are used more efficiently, and better health outcomes are achieved. Thus, personalized medicine may make sense when inefficiencies arise with the application of standard treatments to type 1 patient (*α*(*b* + *d*) < *C*). Notice that if it were possible to identify the type 1 patients, they would never be prescribed the standard treatment as it would be better to leave them untreated. Alternatively, if *α*(*b* + *d*) ≥ *C*, the rationale for the application of personalized medicine would come from higher health benefits yielded by the new treatment.

The new treatment is produced at zero marginal costs by a monopolistic firm. It is assumed that the firm only knows the distribution of prevalence rates across hospitals (e.g. the firm only knows that there is a proportion *q* of high prevalence hospitals and a proportion 1 − *q* of low prevalence hospitals), but ignores the type of hospital is dealing with.

We assume that, without additional information on the patients, all hospitals prefer to apply the standard treatment to all patients rather than to leave them untreated. (*b* − *C* − *π*_*i*_(1 − *α*)(*b* + *d*) ≥ − *d*, *i* = *l*, *h*). There is in the market, at a price *g*, a test that allows to identify, although imperfectly, the type 1 patients. We consider that the test is sold by a firm other than the firm marketing the new treatment. Thus, we do not consider companion diagnostic tests whose price would be set by the same drug manufacturer. Patients receive personalized treatments contingent on the results of the test. The new treatment is applied to the type 1 patients identified by the test and the standard treatment to the type 2 patients.

Let the test be characterized by (*e*, *s*), where *e* ∈ [0, 1] denotes the specificity of the test and *s* ∈ [0, 1] stands for the sensitivity of the test. For simplicity, we assume that the test perfectly classifies the patients: *e* = *s* = 1. In real world, most tests are genetic, and their specificity and sensitivity are quite high. We also assume that *π*_*l*_*b* > *g* (e.g. in the low prevalence hospitals, the aggregated benefits type 1 patients get from the new treatment are above the cost of the test) and *C* > *g* (e.g. the cost of the standard treatment is above the cost of the test).

We consider a sequential decision. First, the firm chooses a pair of prices {*t*_*l*_, *t*_*h*_} for the new treatment to maximize its expected profits. In the second stage, each hospital, given {*t*_*l*_, *t*_*h*_} and the price of the test *g*, decides either to treat all patients with the standard treatment without using the test; or to administer the test, and contingent on the results, to personalize the treatments. Each hospital pursues to maximize the net expected economic benefits, defined as the expected health economic value minus the total costs (treatments plus test).

## Results

### The implementation of personalized medicine under perfect information

Let us first assume that there is no private information on prevalence rates. The firm knows, for each hospital, the prevalence of the condition that causes the disease to the type 1 patients. If the firm wants the hospitals to implement personalize medicine, the price of the new treatment, a key variable influencing the decision, must be subject to some constraints.

When hospital *i*, *i* = *l*, *h*, applies the standard treatment to its patients, the net expected health economic benefits are given by *b* − *C* − *π*_*i*_(1 − *α*)(*b* + *d*). If hospital *i*, *i* = *l*, *h*, applies personalized medicine, the net expected health economic benefits are *π*_*i*_(*b* − *t*_*i*_) + (1 − *π*_*i*_)(*b* − *C*) − *g* = *b* − *C* − *g* − *π*_*i*_(*t*_*i*_ − *C*), where *t*_*i*_ is the price set by the firm. Thus, hospital *i*, *i* = *l*, *h*, prefers to personalize the treatments if and only if:

1 $$ {\pi}_i\left(1-\alpha \right)\left(b+d\right)-g-{\pi}_i\left({t}_i-C\right)\ge 0\Rightarrow \left(1-\alpha \right)\left(b+d\right)+C-\frac{g}{\pi_i}\ge {t}_i\kern1em i=l,h $$

The extra cost of the new treatment (*t*_*i*_ − *C*) must be below the additional benefit (1 − *α*)(*b* + *d*) type 1 patients obtain when they receive the new treatment. Condition (1) sets an upper bound for the price of the new treatment. It easily follows that personalize medicine will not be implemented when this upper bound is negative. Thus, the firm will not sell the new treatment if *π*_*h*_[(1 − *α*)(*b* + *d*) + *C*] ≤ *g*: the extra benefits type 1 patients in the high prevalence hospitals get from the new treatment are lower than the total costs of the test minus the cost of the standard treatment for such patients. As *π*_*h*_*b* > *g*, a necessary condition for this inequality to be satisfied is $\alpha >\frac{c+d}{b+d}$. Intuitively, personalized medicine may not be implemented when the incremental benefits the new treatment provides are relatively low. Notice that type 1 patients would get strictly positive net benefits from the standard treatment, and the rationale for personalized medicine would come from the higher benefits the new treatment provides.

#### Proposition 1:

*Personalized medicine is not implemented if π*_*h*_[(1 − *α*)(*b* + *d*) + *C*] ≤ *g*.

The implementation (at least, partially) of personalized medicine requires *π*_*h*_[(1 − *α*)(*b* + *d*) + *C*] > *g*. From now on, we will suppose that *π*_*l*_[(1 − *α*)(*b* + *d*) + *C*] ≥ *g*. Notice that this condition is always satisfied when $\alpha \le \frac{C+d}{b+d}$, although it may also be fulfilled when $\alpha >\frac{C+d}{b+d}$.

When *π*_*l*_[(1 − *α*)(*b* + *d*) + *C*] < *g* < *π*_*h*_[(1 − *α*)(*b* + *d*) + *C*], personalized medicine will be only implemented by the high prevalence hospitals. In this case, it does not matter whether the information on prevalences is private or public. This case will not be analyzed in the text as it does not provide additional insights. It is available upon request.

If *π*_*l*_[(1 − *α*)(*b* + *d*) + *C*] ≥ *g*, all hospitals implement personalized medicine when the individual prevalence information is publicly shared. From (1), for each *i* = *l*, *h*, the firm chooses the highest feasible price ${t}_i=\left(1-\alpha \right)\left(b+d\right)+C-\frac{g}{\pi_i}$, and its profits are *π*_*i*_*t*_*i*_ = (1 − *α*)(*b* + *d*)*π*_*i*_ + *π*_*i*_*C* − *g*, *i* = *l*, *h*. The firm extracts all surplus, leaving the hospitals indifferent between applying the standard treatment to all patients and personalized medicine.

#### Proposition 2:

Let *π*_*l*_[*b*(1 − *α*) + *C*] ≥ *g. If the firm knows, for each hospital, the prevalence of the condition that causes the disease to the type 1 patients, it charges*${t}_i=b\left(1-\alpha \right)+C-\frac{g}{\pi_i}$., *i* = *l*, *h*, *for the new treatment. Personalized medicine is implemented by all hospitals.*

### The implementation of personalized medicine under asymmetric information

Let us now assume that the firm only knows the distribution of *π* across hospitals. We keep on assuming *π*_*l*_[(1 − *α*)(*b* + *d*) + *C*] ≥ *g* for which implementation of personalized medicine for all types of hospital is feasible.

Under asymmetric information, the firm does not offer the same prices as in the perfect information situation as all hospitals would pay ${t}_l=b\left(1-\alpha \right)+C-\frac{g}{\pi_l}$ and buy *π*_*l*_ treatments. Thus, the firm will not be maximizing its expected profits. The firm has to modify its pricing policy. In order to maximize expected profits, the firm may choose either a pair of prices, one for each type of hospital, such that all hospitals adopt personalized medicine, or a pair of prices such that only the high prevalence hospitals apply personalized treatments. Let us know characterize the optimal pair of prices for both situations.

Let us first assume that the standard treatment is inefficient when applied to type 1 patients, and hospitals prefer to leave them untreated: *α*(*b* + *d*) < *C*. Let {*t*_*l*_, *t*_*h*_} be a pair of prices such that all hospitals adopt personalized treatments. The firm charges a price *t*_*l*_ if the hospital buys *π*_*l*_ treatments, and a price *t*_*h*_ if the hospital buys *π*_*h*_ treatments. These prices must satisfy the participation constraints (e.g. personalized medicine must be preferred to the standard treatment) and the incentive compatibility constraints (e.g. hospitals with prevalence *π*_*i*_ must prefer to pay *t*_*i*_ instead of *t*_*j*_, *i* = *l*, *h* and *i* ≠ *j*).

The participation constraints are:

$$ {\pi}_i\left(b-{t}_i\right)+\left(1-{\pi}_i\right)\left(b-C\right)-g\ge {\pi}_i\left(\alpha b-\left(1-\alpha \right)d-C\right)+\left(1-{\pi}_i\right)\left(b-C\right)\kern1em i=l,h $$

Applying personalized medicine requires first to pay for the test that allows to stratify the patients. Once the patients have been identified, hospital *i* buys *π*_*i*_ new treatments. Benefits from type 1 patients are *π*_*i*_(*b* − *t*_*i*_). Type 2 patients are given the standard treatment yielding benefits (1 − *π*_*i*_)(*b* − *C*). Thus, the left-hand side of the above expression states the net benefits hospital *i* gets when it applies personalized medicine. If hospital *i* applies the standard treatment to all patients, expected benefit from type 1 patients are *π*_*i*_(*αb* − (1 − *α*)*d* − *C*). Notice that the standard treatment cures type 1 patients with probability *α*. Benefits for type 2 patients are (1 − *π*_*i*_)(*b* − *C*). The right-hand side of the above expression states the expected net benefits when hospital *i* applies the standard treatment to all patients. Algebraic manipulation of the above expression yields:

$$ \left(1-\alpha \right)\left(b+d\right)+C-\frac{g}{\pi_i}\ge {t}_i\kern1em i=l,h $$

The incentive compatibility constraint for the high prevalence hospitals is:

$$ {\pi}_h\left(b-{t}_h\right)+\left(1-{\pi}_h\right)\left(b-C\right)-g\ge {\pi}_l\left(b-{t}_l\right)+\left(1-{\pi}_h\right)\left(b-C\right)-g-\left({\pi}_h-{\pi}_l\right)d $$

The left hand side expresses the net economic health benefits from personalized medicine when a high prevalence hospital pays the price *t*_*h*_. If a high prevalence hospital behaves as a low prevalence hospital, it will buy *π*_*l*_ treatments at a price *t*_*l*_, and *π*_*h*_ − *π*_*l*_ type 1 patients will be left untreated, with a loss of (*π*_*h*_ − *π*_*l*_)*d*. Notice that we are assuming that the standard treatment is inefficient for type 1 patients. Algebraic manipulation yields:

$$ \left({\pi}_h-{\pi}_l\right)\left(b+d\right)\ge {\pi}_h{t}_h-{\pi}_l{t}_l $$

The incentive compatibility constraint for the low prevalence hospitals is:

$$ {\pi}_l\left(b-{t}_l\right)+\left(1-{\pi}_l\right)\left(b-C\right)-g\ge {\pi}_l\left(b-{t}_h\right)+\left(1-{\pi}_l\right)\left(b-C\right)-g-\left({\pi}_h-{\pi}_l\right){t}_h $$

The left hand side expresses the net economic health benefits from personalized medicine when a low prevalence hospital pays the price *t*_*l*_. If a low prevalence hospital behaves as a high prevalence hospital, it will buy *π*_*h*_ treatments at a price *t*_*h*_, and *π*_*h*_ − *π*_*l*_ treatments will remain unused. Algebraic manipulation yields:

$$ {\pi}_h{t}_h-{\pi}_l{t}_l\ge 0 $$

If the firm wants all hospitals to adopt personalized medicine, it will select the pair of prices {*t*_*l*_, *t*_*h*_} that solves the following problem:

$$ \underset{\left\{{t}_l,\ {t}_h\right\}}{ \max }q{\pi}_h{t}_h+\left(1-q\right){\pi}_l{t}_l $$

$$ s.t.\kern1em \left(1-\alpha \right)\left(b+d\right)+C-\frac{g}{\pi_i}\ge {t}_i\kern1em i=l,h $$

$$ \left({\pi}_h-{\pi}_l\right)\left(b+d\right)\ge {\pi}_h{t}_h-{\pi}_l{t}_l $$

$$ {\pi}_h{t}_h-{\pi}_l{t}_l\ge 0 $$

In the solution, the participation constraint for the low prevalence hospitals is binding as well as the incentive compatibility constraint for the high prevalence hospitals. Thus:

$$ {t}_l=\left(1-\alpha \right)\left(b+d\right)+C-\frac{g}{\pi_l} $$

$$ \left({\pi}_h-{\pi}_l\right)\left(b+d\right)={\pi}_h{t}_h-{\pi}_l{t}_l\Rightarrow {t}_h=\left(b+d\right)\left(1-\frac{\alpha {\pi}_l}{\pi_h}\right)+\frac{1}{\pi_h}\left({\pi}_lC-g\right) $$

The price of the new treatment for the low prevalence hospitals equals the price the firm would choose if it had symmetric information. These hospitals enjoy no surplus. The price of the new treatment for the high prevalence hospitals is lower than the price under symmetric information. These hospitals get a positive surplus and strictly prefer personalized medicine to the standard treatment. The expected benefits of the firm are:

$$ \begin{array}{l}{B}_{PM}=q{\pi}_h\left[\left(b+d\right)\left(1-\frac{\alpha {\pi}_l}{\pi_h}\right)+\frac{1}{\pi_h}\left({\pi}_lC-g\right)\right]+\left(1-q\right){\pi}_l\left[\left(1-\alpha \right)\left(b+d\right)+C-\frac{g}{\pi_l}\right]\\ {}=\left(b+d\right)\left[\overline{\pi}-\alpha {\pi}_l\Big)\right]-g+{\pi}_lC\end{array} $$

Let us now assume that it is efficient to treat type 1 patients with the standard treatment instead of leaving them untreated: *α*(*b* + *d*) ≥ *C*. In this case, both participation constraints and the incentive compatibility constraint for the low prevalence hospitals do not change. The incentive compatibility constraint for the high prevalence hospitals is now given by:

$$ {\pi}_h\left(b-{t}_h\right)+\left(1-{\pi}_h\right)\left(b-C\right)-g\ge {\pi}_l\left(b-{t}_l\right) + \left(1-{\pi}_h\right)\left(b-C\right)-g+\left({\pi}_h-{\pi}_l\right)\left(\alpha b-\left(1-\alpha \right)d-C\right) $$

If a high prevalence hospital behaves as a low prevalence hospital, it will buy *π*_*l*_ treatments at a price *t*_*l*_ to treat *π*_*l*_ patients and it will apply the standard treatment to both the remaining *π*_*h*_ − *π*_*l*_ type 1 patients and (1 − *π*_*h*_) type 2 patients. Notice than it is now efficient to apply the standard treatment to type 1 patients. Algebraic manipulation of this expression yields:

$$ \left({\pi}_h-{\pi}_l\right)\left[\left(1-\alpha \right)\left(b+d\right)+C\right]\ge {\pi}_h{t}_h-{\pi}_l{t}_l $$

If the firm wants all hospitals to adopt personalized medicine, it will select the pair of prices {*t*_*l*_, *t*_*h*_} that solves the following problem:

$$ \underset{\left\{{t}_l,\;{t}_h\right\}}{ \max }q{\pi}_h{t}_h+\left(1-q\right){\pi}_l{t}_l $$

$$ s.t.\kern1em \left(1-\alpha \right)\left(b+d\right)+C-\frac{g}{\pi_i}\ge {t}_i\kern1em i=l,h $$

$$ \left({\pi}_h-{\pi}_l\right)\left[\left(1-\alpha \right)\left(b+d\right)+C\right]\ge {\pi}_h{t}_h-{\pi}_l{t}_l $$

$$ {\pi}_h{t}_h-{\pi}_l{t}_l\ge 0 $$

As before, the participation constraint for the low prevalence hospitals is binding. By plugging *t*_*l*_ into the incentive compatibility constraint for the high prevalence hospitals, we get:

$$ \begin{array}{c}\hfill \left({\pi}_h-{\pi}_l\right)\left[\left(1-\alpha \right)\left(b+d\right)+C\right]\ge {\pi}_h{t}_h-{\pi}_l\left[\ \left(1-\alpha \right)\left(b+d\right)+C-\frac{g}{\pi_l}\right]\hfill \\ {}\hfill \Downarrow \hfill \\ {}\hfill \left(1-\alpha \right)\left(b+d\right)+C-\frac{g}{\pi_h}\ge {t}_h\hfill \end{array} $$

It turns out that the participation constraint and the incentive compatibility constraint for the high prevalence hospitals are equal. Thus, the expected net health benefits high prevalence hospitals get when they apply the standard treatment to its patients are equal to the expected net health benefits they would get if they behave as low prevalence hospitals. The firm has only to assure the fulfillment of the participation constraints, as it would happen under symmetric information. It follows that the firm chooses the same prices that it would choose when the information on individual prevalence rates is public: ${t}_i=\left(1-\alpha \right)\left(b+d\right)+C-\frac{g}{\pi_l},i=1,2$. This is a surprising result as, under asymmetric information, we should expect the high prevalence hospitals to enjoy an informational rent.

Until now, we have focused on a pricing policy such that both type of hospitals adopt personalized medicine. Alternatively, the firm may follow a price policy such that only the high prevalence hospitals adopt personalized medicine. In this case, the firm may offer one price ${t}_h=\left(1-\alpha \right)\left(b+d\right)+C-\frac{g}{\pi_h}$, and the high prevalence hospitals will be indifferent between personalized medicine and the standard treatment. Notice that the low prevalence hospitals strictly prefer the standard treatment. The expected benefits of the firm would be:

$$ B=q{\pi}_h{t}_h=q{\pi}_h\left[\left(1-\alpha \right)\left(b+d\right)+C\right]-qg $$

After characterizing the optimal pricing policies for each situation, the firm will choose the pricing policy yielding the highest expected profits.

When *α*(*b* + *d*) < *C*, we have:

$$ \begin{array}{c}\hfill B\gtreqless {B}_{PM}\iff q{\pi}_h\left[\left(1-\alpha \right)\left(b+d\right)+C\right]-qg\gtreqless\ \left(b+l\right)\left[\overline{\pi}-\alpha {\pi}_l\Big)\right]-g+{\pi}_lC\hfill \\ {}\hfill \Downarrow \hfill \\ {}\hfill B\gtreqless {B}_{PM}\iff \left(q{\pi}_h-{\pi}_l\right)\left[C-\alpha \left(b+d\right)\right]\ \gtreqless \left(1-q\right)\left[{\pi}_l\left(b+d\right)-g\right]\hfill \end{array} $$

If *qπ*_*h*_ − *π*_*l*_ ≤ 0, full implementation of personalized medicine is optimal for the firm. Otherwise, personalized medicine may be totally or partially implemented. Thus, it may happen that some type 1 patients (those treated in low prevalence hospitals) do not receive personalized treatment.

When *α*(*b* + *d*) ≥ *C*, the firm prefers all hospitals to adopt personalized medicine. Notice that in this case, informational asymmetries are irrelevant, and the firm is better off when it extracts all the surplus from both types of hospitals instead of only from the high prevalence ones.

#### Proposition 3:

*Let α*(*b* + *d*) < *C. Full implementation of personalized medicine requires* (*qπ*_*h*_ − *π*_*l*_)[*C* − *α*(*b* + *d*)] ≤ (1 − *q*)[*π*_*l*_(*b* + *d*) − *g*]*. Otherwise, personalized medicine is only implemented by the high prevalence hospitals. If α*(*b* + *d*) ≥ *C, all hospitals adopt personalized medicine.*

## Discussion

In this paper, we have developed an adverse selection model to describe the behavior of hospitals when they have to decide whether to use personalized medicine to treat a given disease. The model captures in a stylized way the essence of the problem by taking into account the relevant variables and parameters.

In the last decade, personalized medicine has emerged as a novel approach to match new treatments and patients. For some diseases, mainly in the area of oncology, the application of standard treatments has proven to have low therapeutic effects for specific groups of patients. Until recently, there were no mechanisms to stratify the population of patients on the basis of a genetic biomarker, thus precluding the use of specific treatments, if available. Lately, the development of tests (mainly genetics) and therapies has skyrocketed, making feasible the use of personalized medicine.

As it was mentioned in the introduction, most of the economic literature on personalized medicine has focused on the economic evaluation of specific tests and their corresponding new therapies; the results about their efficiency are not conclusive and the strength of evidence available for existing personalized medicine technology varies widely, as some authors have remarked [17, 18].

Theoretical studies on the economics of personalized medicine are less common as it was aforementioned at the introduction section. As their goals and methodological approaches were different from ours, their results are not directly comparable. Our analysis has pursued to present in an integrated way some of the relevant issues that should be taking into account to make decisions on the implementation of personalized medicine. In real world practice, exact figures of the parameters would be needed to populate the model so that it yield final results to adopt the decision.

The model was developed for a health system with free pricing for new treatments, without including an authorization process. In some jurisdictions with public systems of health insurance, a central health authority defines the price and reimbursement policy. Our model allows to accommodate this fact by implicitly assuming that the firm may set for the new treatment any price below a price ceiling fixed by the central health authority in its commercial relationships with hospitals. Likewise, drug prices may be common to all hospitals or each hospital may negotiate the price with the manufacturer. Our model considers both scenarios. We have focused on the situation in which hospitals may end up paying different prices for the same treatment as a result of the existence of asymmetric information between the hospital and the manufacturer.

For the sake of simplicity we have considered that that the test perfectly classifies the patients. In real world, the specificity and sensitivity of the tests are usually below one, and this may influence the adoption of personalized medicine as expected health benefits and costs are affected by these parameters. Within the framework of the model, from a qualitative point of view, there would be no change in the analysis, although the specific conditions for the adoption of personalized medicine would be different as well as the prices for the new treatment fixed by the firm.

We have also assumed, for the sake of mathematical simplicity, that all patients benefit from the received treatments although patients are better off when they are matched with the right treatment according to their characteristics. Although we are aware of the existence in real world of medical uncertainty on the right treatments and their efficacy for many diseases, we have opted for the simplest formulation of the problem at hand to highlight the key issues. A more sophisticated model should incorporate these aspects into the analysis. Although promising, there are uncertainties regarding the efficacy of treatments and relative sizes of target populations that demand further research as stated by Towse and Garrison [25], O’Donnell [26] and Faulkner et al. [27].

## Conclusions

From the perspective of the health system, it is better not to reveal individual prevalence information if standard treatments are inefficient when applied to unresponsive patients. Otherwise, informational asymmetry on the prevalence rates is irrelevant. This is a surprising result as private information should be valuable. Personalized medicine may be fully or partially adopted depending on the proportion of high prevalence hospitals.

When informational asymmetries matter, hospitals with high prevalence benefit from the private information by paying for the new treatment a price lower than the price they pay when prevalence information is public. For the low prevalence hospitals, asymmetric information does not make a difference on the price.

If one were to use the model framework to adopt decisions on the implementation of personalized medicine more accurate statistical and economic information related to tests results and efficacy of the treatments would be needed. In general, the efficiency of personalize medicine is still waiting for assessment. Although personalized medicine was thought to be the panacea for patients and healthcare systems, except on oncology, it is still far from being extensively applied.
